# Fellow-Workers and the Roll of Honour

**Published:** 1915-08-28

**Authors:** 


### ROUND THE WORLD.

[The Editor invites Early Information and Contributions Marked "Fellow-Workers."]

### Fellow-Workers and the Roll of Honour.

Lieutenant John Cornock Hawkes, R.A.M.C., killed in Flanders, was an Irishman who qualified by taking the Scottish "Triple" about five years ago. Taking one of the temporary R.A.M.C. commissions just before Christmas, he had lately been attached to the King's Royal Rifle Corps (8th Battalion).

The use of chlorine gas as a military weapon has naturally led to immediate investigations of its effects on animal tissues, in which connection Sir Edward Albert Schäfer has communicated some remarkably interesting observations to the *British Medical Journal* (July 14, 1915), considerably adding to our knowledge of what may be expected to follow its inhalation. A physiologist of world-renown, Sir Edward Schäfer will doubtless be listened to with deference even in this unusual field of pharmacological experiment. Formerly Jodrell professor of physiology at University College, he has for some years occupied the chair of physiology at Edinburgh. The possessor of numerous honorary degrees and other academic distinctions, this celebrated scientist became medically "qualified" in 1874, when he took the diploma of M.R.C.S. Eng.

The following names of practitioners in the Poor-Law Service have to be added to the list of those medical officers of health, tuberculosis officers, and school doctors who have joined the R.A.M.C., and who have not so far been mentioned in these notes: Captain Cuthbert Blundell Moss-Blundell, M.D. Brux., M.R.C.S. Eng., D.P.H. Camb., an old student of University College and St. Thomas's Hospital, who occupies the posts of medical officer of health, tuberculosis officer, and school medical officer for Huntingdon County; Major Walter Linney Hawksley, M.B., Ch.B. Liverp., D.P.H., of the 2nd West Lancashire Field Ambulance, who in civil life is assistant medical officer of health for Liverpool; Lieutenant-Colonel William Albert Wetwan, M.R.C.S., L.S.A., an old London Hospital man, who has for some years been medical officer of health at Bridlington; also Lieutenant-Colonel Francis Edward Fremantle, medical officer of health for Herts, who is a distinguished representative of both Guy's and "Bart.'s," and is an M.B., M.Ch. Oxon., and D.P.H., as well as being a Fellow of both Royal Colleges.

Also the following, who have all, it is understood, taken commissions in the R.A.M.C. as lieutenants: Wilfred Barkes, M.D., B.S. Durh., medical officer of health for Houghton-le-Spring (R.D.); George Spencer Armitage Bishop, M.B., Ch.B. Liverp., medical officer Asfordby district of the Melton Mowbray Union; Ernest Francis Clowes, M.R.C.S., L.R.C.P. (Guy's), medical officer and public vaccinator at Dursley; Fritz Salo Eschwege, M.R.C.S., L.R.C.P. (Cambridge and London Hospital), medical officer and public vaccinator at East Grinstead; Arthur Edward Lyster, J.P., M.D. Brux., M.R.C.S. Eng., medical officer at Chelmsford, who is also medical superintendent Essex Royal Memorial Association for the Treatment of Consumption, and medical superintendent of the Alfred Boyd Memorial Tuberculosis Sanatorium; Butler Hogan, L.R.C.P. Irel., L.S.A. (Dublin and London Hospital), parish medical officer for Stepney Union; and Vivian Gray Maitland, M.R.C.S., L.R.C.P., D.P.H. Dub. (University of Birmingham), medical officer of health for Nuneaton.

Lieutenant John Cattanach, R.A.M.C., who has died of wounds, took up his commission last October, and was eventually sent out to the Dardanelles. An Edinburgh student, who distinguished himself as an athlete, he graduated M.B., B.Ch. in 1912. Mr. Cattanach was a Scottish international player for both hockey and athletics, whilst he represented Edinburgh at the inter-university sports for several years.

Second-Lieutenant Bryan Osmond Dewes (Middlesex Regiment) had entered the medical school at St. Thomas's Hospital with a scholarship not long before the outbreak of war. Enlisting in the Artists' Rifles almost immediately, he was given a commission in February of this year, and before his death had done good work with a machine-gun section.

Colonel Neville Manders, whose name recently appeared in a Dardanelles casualty list amongst those of officers killed, was a distinguished officer of the Army Medical Service attached to the staff of the Australian and New Zealand troops. He qualified M.R.C.S. Eng. in 1883.

The *St. Thomas's Hospital Gazette* follows the excellent practice of publishing lists of St. Thomas's men who have lately gone to serve with the R.A.M.C., and adds the following names to those previously given: Cecil Bennett, W. K. Bigger, G. A. Bird, A. P. Bowdler, G. N. Brandon, W. Burt, H. H. Carleton, H. H. R. Clarke, W. Deane, J. H. Drew, J. N. F. Ferguson, H. D. Field, C. F. Harford, W. Harmens, J. H. Hart, R. Hughes, A. C. Inman, G. D. Kerr, G. E. Kinnersley, C. L. G. Powell, R. T. Raine, W. F. Rhodes, F. B. G. Stableford, A. J. C. Tingey, E. H. Walker, J. Douglas Wilkinson, and G. R. C. Wilson.

Amongst medical students who have died as combatants must also be included Lieutenant R. M. Stewart Boyd, who, although in his final year at Glasgow, accepted a commission in the Highland Light Infantry and was eventually sent to the East. Also Second-Lieutenant Thomas Erskine Baillie, of the Argyll and Sutherland Highlanders, another Glasgow student, who joined the Army in the early days of the war.

Mr. Frank Cole Madden, who has lately been appointed operating surgeon to the British Forces in Egypt, is a well-known Cairo practitioner. An M.D. Melbourne, F.R.C.S. Eng., Mr. Madden, whom it is understood will be attached to the military hospital at Cairo Citadel, is professor of surgery in the Egyptian Government School of Medicine, and senior surgeon to the Kasr-el-Ainy Hospital, Cairo.

